# Supplementary material for: Uncovering spin-orbit coupling-independent hidden spin polarization of energy bands in antiferromagnets
Source: Nat Commun. 2023 Aug 31;14:5301. doi: 10.1038/s41467-023-40877-8 (PMC10471643; doi:10.1038/s41467-023-40877-8)
Supplement: Supplementary file 1 — Supplementary information [file 41467_2023_40877_MOESM1_ESM.pdf]

Supplementary Information

Uncovering spin-orbit coupling-independent hidden spin polarization of energy bands in antiferromagnets

Lin-Ding Yuan[1], Xiuwen Zhang[1], Carlos Mera Acosta[2], Alex Zunger[1]

[1]Renewable and Sustainable Energy Institute, University of Colorado, Boulder, Colorado 80309, USA

[2]Center for Natural and Human Sciences, Federal University of ABC, Santo Andre, São Paulo, Brazil

A. Symmetry conditions for SOC-independent hidden spin polarization in collinear AFM

The hidden spin polarization in the absence of SOC arises in AFM, when the bulk symmetry enforces spin degeneracy of the band while the sector symmetry allows spin splitting.

The bulk symmetry of a spin degenerate antiferromagnetic compound needs to preserve either ΘIT or UT symmetry. Depending on the which of the two symmetries ΘIT and UT are preserved spin degenerate bulk AFM can be divided into three prototypes: (1) Bulk AFM compounds that violate UT but preserves ΘIT symmetry which is referred to as spin splitting prototype 1 (SST-1) bulk; (2) Bulk AFM compounds that preserve both UT and ΘIT symmetry which is referred to as SST-2 bulk; (3) Bulk AFM compounds that preserve UT but violate ΘIT symmetry which is referred to as SST-3 bulk.

The sector symmetry of a spin split sector needs to violate both UT and ΘIT symmetry. Depending on the magnetic type of the constitute sector of the bulk AFM the spin split sector can be either (4) AFM sector that violate both UT and ΘIT symmetry which is referred to as SST-4 sector; or (5) FM (also include Ferrimagnets (FiM)) sector that violate both UT and ΘIT symmetry which is referred to as SST-5 sector. The two spin split sector prototypes correspond to the two SOC-independent mechanisms for spin splitting, that is (a) magnetic mechanism that create spin splitting even in the absence of SOC in antiferromagnetic compounds; and (b) Zeeman mechanism of the FM sector with non-zero intrinsic magnetic moment.

B. Band structure of tetragonal CuMnAs

To compare with previously reported band structure results. In addition to Figure 3(c) in the main text, we calculated the band structure along the complete conventional momentum space path. The results are presented in Figure S1, which agrees well with those reported[1,2].

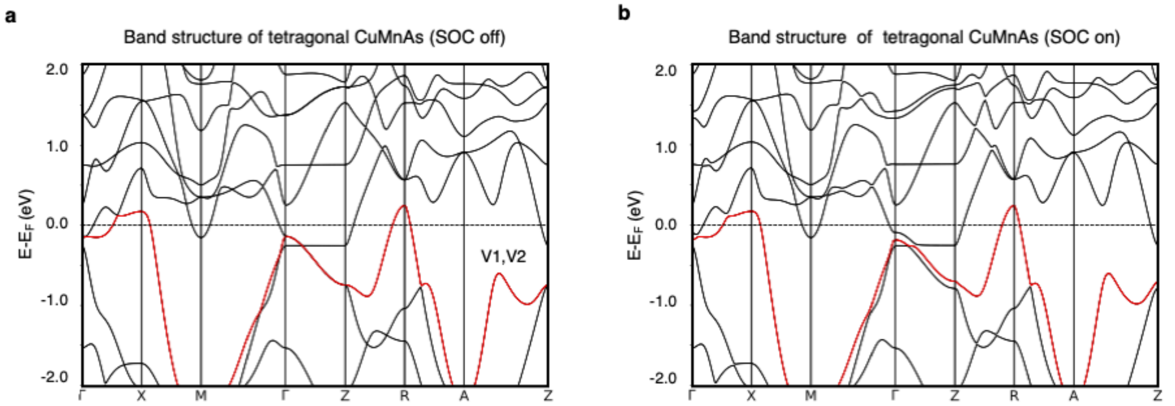

Figure S1: Energy spectrum of antiferromagnetic tetragonal CuMnAs on the conventional k-paths. a with SOC turned off, and **b** with SOC turned on.

C. Additional examples of SOC-independent hidden spin polarization in collinear antiferromagnets with DFT results

Figure S2 illustrates the "hidden spin polarization" effect in hexagonal $CoBr_2$[3] being bulk SST-2 class (MSG Cc2/c) but made of SST-5 class FM $CoBr_2$ layers (α-sector and β-sector in Fig. S2a). The crystal is antiferromagnetically ordered with its magnetic moments collinearly aligned in (001) direction. The two $FeBr_2$ layers are connected by both the ΘIT and the UT symmetry which restores the spin degeneracy of the bulk and results in a compensated net spin polarization (Fig. S2b). However, the corresponding spin polarization for the bottom two conduction bands (C1 and C2) projected onto α-sector and β-sector (hidden spin polarization), shown in Fig. S2c, are non-zero and compensate each other.

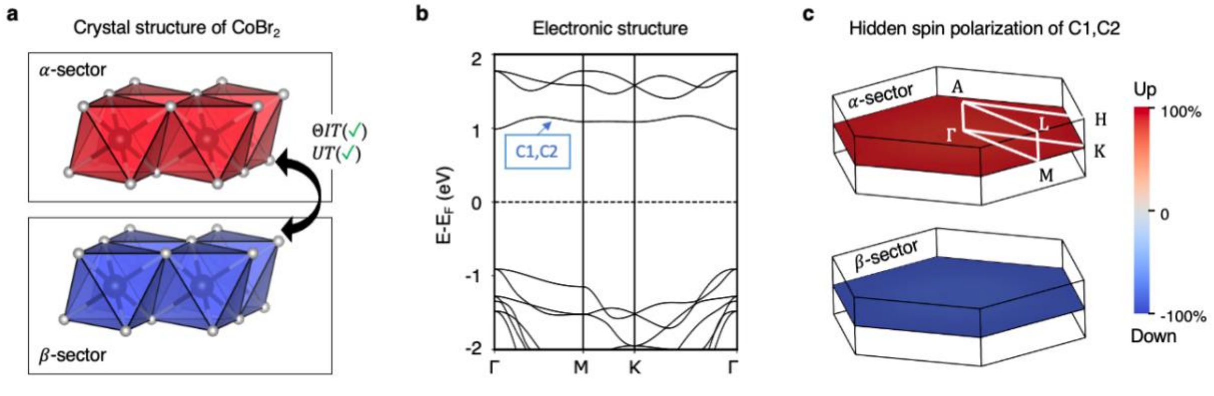

Figure S2: Hidden spin polarization from individual ferromagnetic sectors in bulk hexagonal $CoBr_2$ (bulk belonging to SST-2 class with sector belonging to SST-5 class). a crystal structure of antiferromagnetic $CoBr_2$ composed of two ferromagnetic layers with opposite magnetization (indicated by red and blue polyhedra) in the unit cell. The two layers are referred as sector-α and sector-β, respectively; **b** spin degenerate bands of $CoBr_2$; **c** Hidden spin polarization from each individual sector of the lowest two conduction bands (C1 and C2) on ΓMK k-plane. The up and down spins are mapped to the color from blue

to red. The crystal and magnetic structure for hexagonal $CoBr_2$ used in our DFT calculations are taken from Ref. [3]. The electronic structure and hidden spin polarization are calculated using the PBE+U method with U=3.32 eV, J=0 eV on Co-3d orbits.

Figure S3 illustrates the "hidden spin polarization" effect in tetragonal $Ca_3Ru_2O_7$ [4] being bulk SST-3 class (MSG Pcna2₁) but made of SST-5 class FM RuO_7 sectors (α-sector and β-sector in Fig. S3a). The crystal is antiferromagnetically ordered with its magnetic moments collinearly aligned in (010) direction. The two ferromagnetically ordered Ru_2O_7 sectors are connected by the UT symmetry which restores the spin degeneracy of the bulk and results in a compensated net spin polarization (Fig. S3b). However, the corresponding spin polarization for the bottom two conduction bands (C1 and C2) projected onto α-sector and β-sector (hidden spin polarization), shown in Fig. S3c, are nonzero and compensate each other.

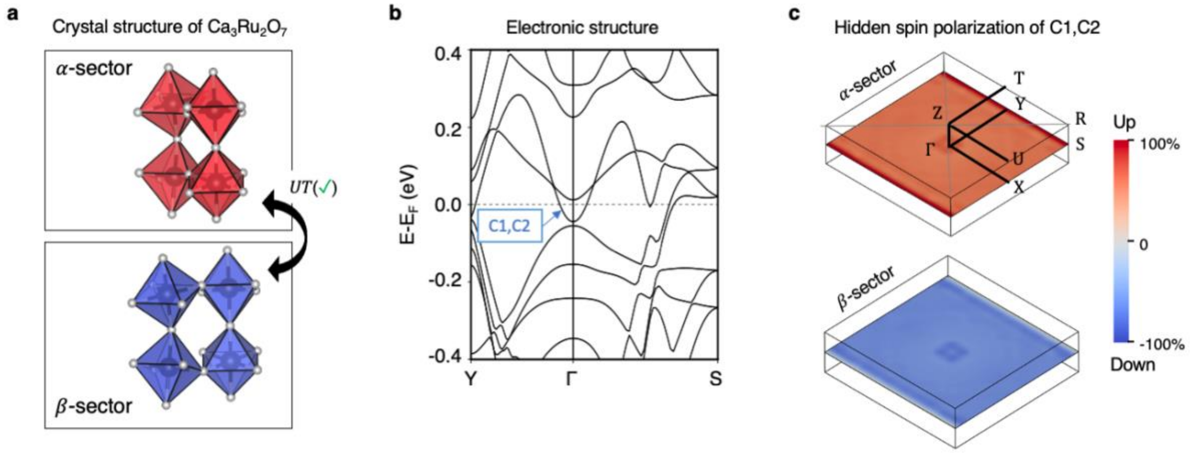

Figure S3: Hidden spin polarization from individual ferromagnetic sectors in bulk orthorhombic $Ca_3Ru_2O_7$ (bulk belonging to SST-3 class with sector belonging to SST-5 class). a crystal structure of antiferromagnetic $Ca_3Ru_2O_7$ composed of two ferromagnetic sectors with opposite magnetization (indicated by red and blue polyhedra) in the unit cell. The Ca atoms are dismissed. The two sectors are referred as sector-α and sector-β, respectively; **b** spin degenerate bands of $Ca_3Ru_2O_7$; **c** Hidden spin polarization from each individual sector of the lowest two conduction bands (C1 and C2) on ΓXS k-plane. The up and down spins are mapped to the color from blue to red. The crystal and magnetic structure for tetragonal $Ca_3Ru_2O_7$ used in our DFT calculations are taken from Ref. [4]. The electronic structure and hidden spin polarization are calculated using the GGA functional without U.

Figure S4 illustrates the "hidden spin polarization" effect in La_2NiO_4 [5] being bulk SST-2 class (MSG: Pcmna) but made of SST-4 class antiferromagnetic NiO_6 layers (α-sector and β-sector in Fig. S4a). The crystal is antiferromagnetically ordered with its magnetic moments collinearly aligned in (100) direction. The two antiferromagnetically ordered NiO_6 sectors are connected by both the ΘIT and UT symmetry which restores the spin degeneracy of the bulk and results in a compensated net spin polarization (Fig. S4b). However, the corresponding spin polarization for

the top two valence bands (V1 and V2) projected onto α-sector and β-sector (hidden spin polarization), shown in Fig. S4c, are nonzero and compensate each other.

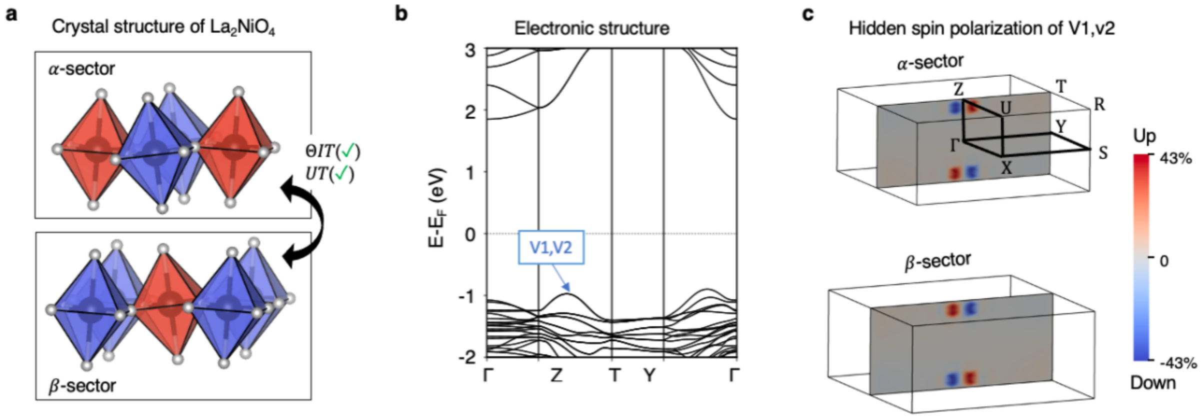

Figure S4: Hidden spin polarization from individual antiferromagnetic sectors in bulk La_2NiO_4 (bulk belonging to SST-2 class with sector belonging to SST-4 class). a crystal structure of antiferromagnetic La_2NiO_4 composed of two antiferromagnetic sectors with opposite magnetic ordering (indicated by red and blue polyhedra) in the unit cell. The two layers are referred as sector-α and sector-β, respectively; **b** spin degenerate bands of La_2NiO_4. **c** Hidden spin polarization from each individual sector of the highest two valence bands (V1 and V2) on ΓYT k-plane. The up and down spins are mapped to the color from blue to red. The crystal and magnetic structure for tetragonal La_2NiO_4 used in our DFT calculations are taken from Ref. [5]. The electronic structure and hidden spin polarization are calculated using the PBE+U method with U=5.0 eV, J=0 eV on Mn-3d orbits.

Figure S5 illustrates the "hidden spin polarization" effect in MnS_2 [6] being bulk SST-3 class (MSG: Pcmna) but made of SST-4 class antiferromagnetic MnS_6 layers (α-sector and β-sector in Fig. S5a). The crystal is antiferromagnetically ordered with its magnetic moments collinearly aligned in (010) direction. The two antiferromagnetically ordered MnS_6 sectors are connected by the UT symmetry which restores the spin degeneracy of the bulk and results in a compensated net spin polarization (Fig. S5b). However, the corresponding spin polarization for the top two valence bands (V1 and V2) projected onto α-sector and β-sector (hidden spin polarization), shown in Fig. S5c, are nonzero and compensate each other. Unlike the other cases, the hidden spin polarization shown in Fig. S5c is very small. This is because the two sectors are spatially close to each other, therefore strongly coupled to each other leading to a highly compensated spin polarization.

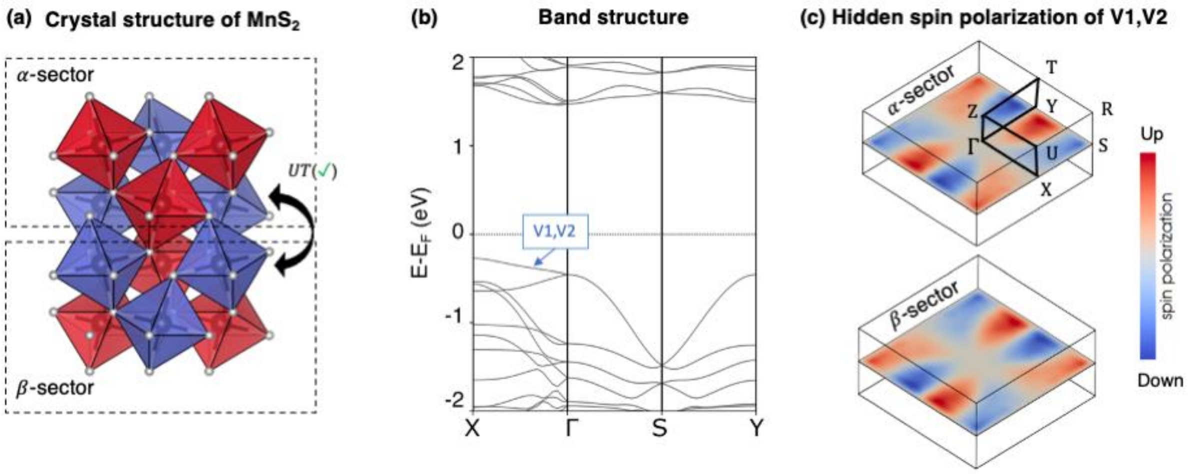

Figure S5: Hidden spin polarization from individual antiferromagnetic sectors in bulk MnS_2 (bulk belonging to SST-3 class with sector belonging to SST-4 class). a crystal structure of antiferromagnetic MnS_2 composed of two antiferromagnetic sectors with opposite magnetic ordering (indicated by red and blue polyhedra) in the unit cell. The two layers are referred as sector-α and sector-β, respectively; **b** spin degenerate bands of MnS_2. **c** Hidden spin polarization from each individual sector of the highest two valence bands (V1 and V2) on ΓXS k-plane. The up and down spins are mapped to the color from blue to red. The crystal and magnetic structure for tetragonal MnS2 used in our DFT calculations are taken from Ref. [6]. The electronic structure and hidden spin polarization are calculated using the PBE+U method with U=5.0 eV, J=0 eV on Mn-3d orbits.

D. Effect of external electric field on FeSe – a SOC-independent hidden spin polarized antiferromagnetic material constructed from 2D layered magnets

Figure S6 illustrates how SST-4 class monolayers (with SS) are ΘIT-asymmetric subsets forming an SST-1 class bilayer that is ΘIT-symmetric (without SS), i.e., spin polarization at different SST-4 class sectors is mutually compensated by means of the stacking of layers that restores the ΘIT symmetry. The DFT band structure calculations for the SST-4 class FeSe monolayer reveals a spin splitting of about 5 meV near the Fermi energy (Fig. S6a). This spin splitting is the result of the antiferromagnetic configuration with two different local environments in the unit cell (dotted line) breaking the ΘIT and UT symmetries. When FeSe monolayers interact via the Van der Waals potential to form a bilayer with layer exactly equivalent (i.e., connected through the ΘIT symmetry), the FeSe bilayer belongs to the SST-1 class. As a consequence, the band structure is completely spin degenerated (Fig. S6b). The compensation between FeSe layers in the bilayer system explicitly demonstrates the concept of the hidden AFM-SS. Remarkable, the bands in the ΘIT-symmetric bilayer have hidden spin-polarization, where spin bands are formed by orbitals spatially localized at different ΘIT-asymmetric subsets (Fig. S6b). As expected, an external electric field breaks the ΘIT symmetry, leading then to manifestation of the hidden spin

polarization in a bilayer with ΘIT-asymmetric subsets that compensate each other. The SS magnitude depends on the intensity of the external electric field. Different from the relativistic Rashba and Dresselhaus SS that require the SOC, the electric field induces in the SST-1 FeSe bilayer a non-zero SS even in the absence of SOC (Fig. S6c).

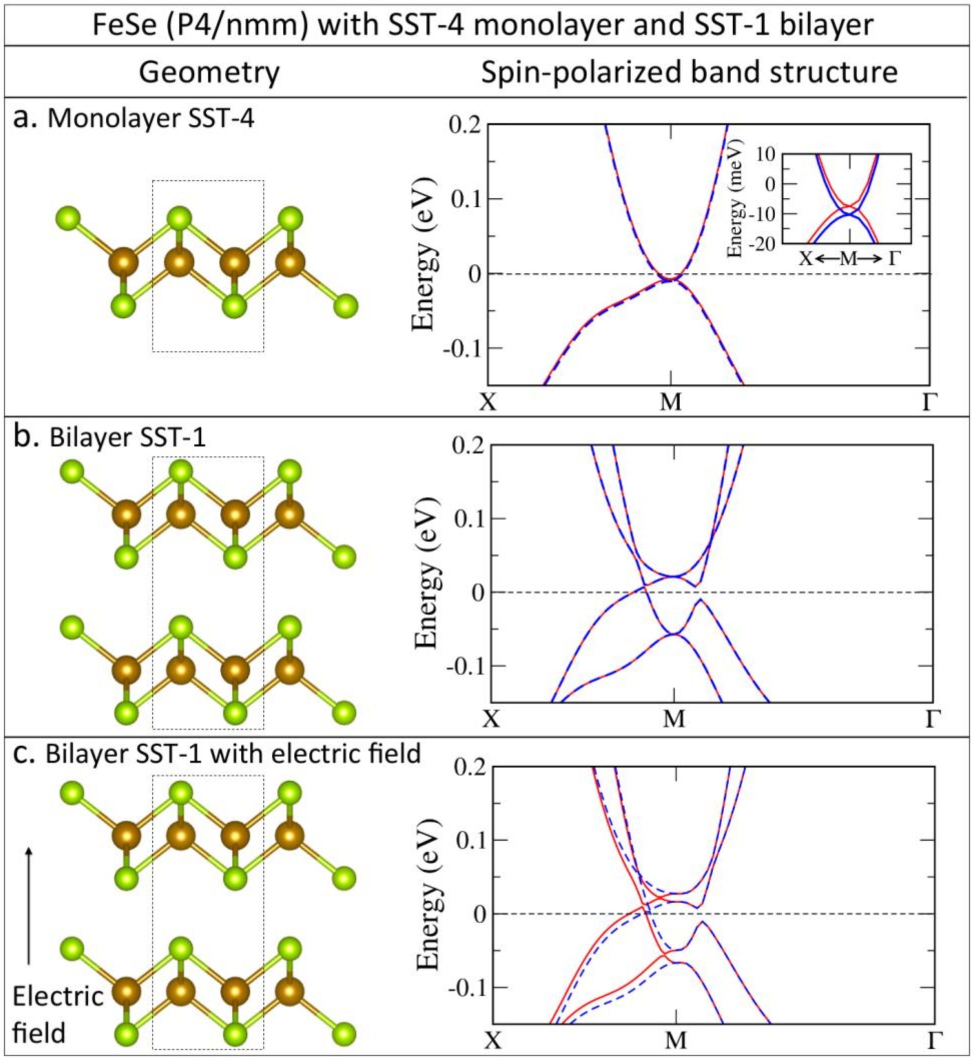

Figure S6: Geometry and DFT band structure calculation for an illustrative example of compensation between SST-4 2D monolayers forming a SST-1 2D bilayer. a SST-4 FeSe monolayer with SS of about 5 meV near the Fermi energy; **b** SST-1 FeSe bilayer with no SS, and **c** SST-1 FeSe bilayer with an external electric field of 0.3 eV/Ang. All DFT+U calculations are performed in the absence of SOC and considering only collinear local magnetic moments. While the SS vanishes when the two SST-4 are connected by the ΘIT symmetry in the bilayer, the external effective field breaks the inversion symmetry, and hence, the ΘIT symmetry. Even in the absence of SOC, the electric field induced a non-zero SS along all symmetry paths in the Brillouin zone. Red and blue lines stand for bands with up and down spin, respectively.

References

1 Veis, M. *et al.* Band structure of CuMnAs probed by optical and photoemission spectroscopy. *Physical Review B* **97**, 125109, doi:10.1103/PhysRevB.97.125109 (2018).

2 Xu, S.-G. *et al.* Dirac fermions in the antiferromagnetic spintronics material CuMnAs. *Physical Review B* **102**, 125123, doi:10.1103/PhysRevB.102.125123 (2020).

3 Wilkinson, M. K., Cable, J. W., Wollan, E. O. & Koehler, W. C. Neutron Diffraction Investigations of the Magnetic Ordering in FeBr2, CoBr2, FeCl2, and CoCl2. *Physical Review* **113**, 497-507, doi:10.1103/PhysRev.113.497 (1959).

4 Yoshida, Y. *et al.* Crystal and magnetic structure of Ca3Ru2O7. *Physical Review B* **72**, 054412, doi:10.1103/PhysRevB.72.054412 (2005).

5 Rodriguez-Carvajal, J., Fernandez-Diaz, M. T. & Martinez, J. L. Neutron diffraction study on structural and magnetic properties of La2NiO4. *Journal of Physics: Condensed Matter* **3**, 3215, doi:10.1088/0953-8984/3/19/002 (1991).

6 Corliss, L. M., Elliott, N. & Hastings, J. M. Antiferromagnetic Structures of MnS2, MnSe2, and MnTe2. *Journal of Applied Physics* **29**, 391-392, doi:10.1063/1.1723149 (1958).

7 Mounet, N. *et al.* Two-dimensional materials from high-throughput computational exfoliation of experimentally known compounds. *Nature Nanotechnology* **13**, 246-252, doi:10.1038/s41565-017-0035-5 (2018).
